# Reply to: comments on "The effects of norepinephrine in shockable cardiac arrest, a scoping review"

**DOI:** 10.1186/s13049-026-01569-6

**Published:** 2026-02-16

**Authors:** S. J. M. Bouman, E. Baldussu, G. H. L. M. Franssen, G. J. van Geffen, J. Bruhn, C. Slagt, L. P. W. Mommers

**Affiliations:** 1 Department of Anaesthesiology and Pain Medicine, Maastricht University Medical Centre, Maastricht, the Netherlands; 2 Emergency Medical Services, Gelderland-Zuid, Netherlands; 3 Information Specialist Department of Education, Content and Support, University Library, Maastricht University, Maastricht, Netherlands; 4 Helicopter Emergency Medical Service Lifeliner 3, Nijmegen, the Netherlands; 5 Department of Anaesthesiology, Pain and Palliative Medicine, Radboud University Medical Centre, Nijmegen, the Netherlands

We thank *Camp et.al.* [1] for their thoughtful remarks on our scoping review concerning norepinephrine use in shockable cardiac arrest [2]. Their critical assessment illustrates how open scientific dialogue can help refine hypotheses and move from pathophysiological plausibility or experimental signals towards robust clinical evidence and guideline change.

## Appreciation of scientific debate

The comment raises key questions regarding study selection, interpretation of outcomes and the clinical implications of the findings of the scoping review. Engaging these remarks is essential to clarify the scope and limitations of our work, and to prevent premature or overextended conclusions being drawn from a heterogeneous and largely preclinical evidence base. In that sense, the letter constructively contributes to the central aim of both papers: to explore whether norepinephrine might offer potential advantages during cardiac arrest resuscitation, acknowledging that epinephrine remains the currend guideline-mandated vasopressor.

## Scope and inclusion of historical and lower-quality evidence

By design, a scoping review aims to map and characterize the available body of evidence, rather than provide a narrow, high‑certainty effect estimate as in a systematic review or meta‑analysis. It is therefore inherent to this methodology that both older and methodologically diverse studies are identified and described, particularly in topics where contemporary randomized human data are lacking.

In our review, we deliberately chose to extend inclusion beyond the randomized controlled trials in humans. We opted for a broad inclusion of studies, including animal models and lower-level clinical studies, such as case reports, provided they reported on the administration of norepinephrine during shockable cardiac arrest. This broad approach was adopted to obtain the most comprehensive overview of the potential physiological and clinical effects of norepinephrine in this specific context.

Including animal studies and case reports does not imply equivalence with high‑quality human randomized trials. Yet such studies can still be valuable; particularly in the search for alternative pharmacological strategies in a research-difficult clinical context. Observational studies may help identify mechanistic pathways or hemodynamic profiles that warrant further investigation. Historically, animal studies [3–5] were instrumental in the initial adoption of epinephrine into cardiac arrest guidelines across the 1980s [6] in the United States of America and the 1990s in Europe [7].

## Rationale for exploring alternative vasopressor strategies

The central motivation for our review was that epinephrine – despite large and high quality studies—did not translated into consistent improvements in longer‑term neurological outcome [8–12]. Recent literature underscores the importance of optimizing intra‑arrest hemodynamics [13], including coronary perfusion pressure and myocardial blood flow, to improve defibrillation success and reduce the risk of re‑arrest. Norepinephrine has been suggested as a potentially alternative.

Given that current ALS guidelines recommend epinephrine and do not advocate norepinephrine as standard vasopressor during cardiac arrest, any search for alternative strategies must start from a lower‑level evidence base. In that context, animal experiments, observational human data and case reports can provide incremental insights into whether intra‑arrest norepinephrine is physiologically plausible and warrant further clinical research.

## Aim of the review and position of the epinephrine comparison

Our explicit aim was not to perform a head‑to‑head comparative effectiveness analysis of norepinephrine versus epinephrine, neither to challenge existing guideline recommendations. Instead, the primary goal was to describe the effects of norepinephrine when administered during shockable cardiac arrest, drawing on all available studies that met our broad inclusion criteria.

Given that epinephrine is the guideline‑mandated standard vasopressor during cardiopulmonary resuscitation, it was considered clinically relevant to briefly situate norepinephrine within this existing therapeutic landscape. Therefore, a short comparative note was included, indicating that norepinephrine appears to offer potential advantages over epinephrine regarding certain hemodynamic parameters and might be associated with favourable signals in terms of re‑arrest and possibly survival as derived of animal studies with some supportive human data.

This comparative remark was not intended as a definitive statement of superiority, but as a concise synthesis of the patterns observed across the heterogeneous body of evidence, framed by the explicit statement that the overall level of evidence is limited and that firm conclusions cannot be drawn.

## Neurological outcome, cerebral perfusion and post‑ROSC meta‑analysis

The comment rightly emphasizes the importance of neurological outcome as the most clinically relevant endpoint in cardiac arrest research. In the scoping review, direct long-term neurological consequences of norepinephrine use during cardiac arrest were reported only sparsely and in varying ways. However, several included animal studies did evaluate cerebral perfusion and cerebral blood flow in relation to norepinephrine administration during cardiac arrest. These studies consistently reported improved or preserved cerebral hemodynamic parameters compared with intra‑arrest baseline or comparator conditions.

While these data are preclinical and cannot be equated with functional neurological outcome in humans, they do address a mechanistic intermediate endpoint: cerebral perfusion. This aspect is biologically linked to neurological recovery. Again, from the perspective of hypothesis generation, these findings were considered relevant to mention in the context of potential neurological implications.

The meta‑analysis cited by the commenting authors focuses on norepinephrine versus epinephrine in the management of post‑ROSC shock [14]. A selected population of patients who have already achieved ROSC and who are in a different hemodynamic and pathophysiological phase of the cardiac arrest continuum. The primary outcomes in the referred meta‑analysis include survival and recurrence of cardiac arrest in the post-cardiac-arrest-shock setting, not the use of norepinephrine during the arrest itself. The scoping review referenced this meta-analysis in the discussion section, predominantly in the context of re‑arrest and arrhythmia risk after ROSC, rather than as direct evidence regarding neurological outcomes associated with intra‑arrest norepinephrine administration. Extrapolating post‑ROSC vasoactive data into the intra‑arrest setting with more heterogenous patient groups and with different hemodynamic profiles, would in our view be scientifically problematic.

The animal studies included in the scoping review specifically administered norepinephrine administration during cardiac arrest and measured cerebral perfusion indices under those conditions. Although these data cannot substitute for human neurological outcome trials, they are directly aligned with the intra‑arrest research question that motivated our review.

## Clinical implications and need for further research

Overall, the available evidence suggests that norepinephrine administration during shockable cardiac arrest may offer benefits in terms of macrocirculatory and myocardial hemodynamics, cerebral blood flow, potentially survival and re-arrest. These findings are based on animal data with limited and heterogeneous support from human studies. At the same time, the quality, size and design of the included studies do not allow any meta-analyses or firm recommendations to change current medication protocols.

From the authors’ perspective and in addition to the conclusion of the scoping review, the most appropriate translation of the current evidence is therefore: to recognize norepinephrine as a physiologically plausible and experimentally supported candidate vasopressor in shockable arrest. We emphasize that more human studies are essential before any changes to clinical protocols can be considered. Concurrently, evidence from clinical practice suggests that the standard resuscitation protocol may be inadequate for certain patients, in whom return of spontaneous circulation is achieved after deviation from the standard protocol [15].

In summary, the concerns raised in the comment align with the cautious interpretation stated in the scoping review. The authors remain convinced that a broad, scoping approach—including older, preclinical and lower‑level human evidence— is appropriate at this exploratory stage. Identifying mechanistic or hemodynamic signals within such data is an important first step before higher‑quality clinical trials can be designed. Intra‑arrest norepinephrine usage merits further investigation, while current guideline‑recommended use of epinephrine remains unchanged pending stronger data.

## Data Availability

No datasets were generated or analysed during the current study.
